# Application of Machine Learning in Chronic Kidney Disease: Current Status and Future Prospects

**DOI:** 10.3390/biomedicines12030568

**Published:** 2024-03-03

**Authors:** Charlotte Delrue, Sander De Bruyne, Marijn M. Speeckaert

**Affiliations:** 1 Department of Nephrology, Ghent University Hospital, 9000 Ghent, Belgium; charlotte.delrue@ugent.be; 2 Department of Laboratory Medicine, Ghent University Hospital, 9000 Ghent, Belgium; sander.debruyne@uzgent.be; 3 Research Foundation-Flanders (FWO), 1000 Brussels, Belgium

**Keywords:** machine learning, chronic kidney disease, diabetic nephropathy, IgA nephropathy, hemodialysis

## Abstract

The emergence of artificial intelligence and machine learning (ML) has revolutionized the landscape of clinical medicine, offering opportunities to improve medical practice and research. This narrative review explores the current status and prospects of applying ML to chronic kidney disease (CKD). ML, at the intersection of statistics and computer science, enables computers to derive insights from extensive datasets, thereby presenting an interesting landscape for constructing statistical models and improving data interpretation. The integration of ML into clinical algorithms aims to increase efficiency and promote its adoption as a standard approach to data interpretation in nephrology. As the field of ML continues to evolve, collaboration between clinicians and data scientists is essential for defining data-sharing and usage policies, ultimately contributing to the advancement of precision diagnostics and personalized medicine in the context of CKD.

## 1. Machine Learning

The emergence of artificial intelligence (AI) has led to machines increasingly performing complex tasks with impressive outcomes. Within the context of medicine, machine learning (ML) stands out as the most promising aspect of AI and is poised to become an element of daily medical routines. Consequently, it is important for physicians to familiarize themselves with ML and AI and view them as facilitators rather than adversaries. Machines are already enhancing the decision-making processes of physicians [1]. ML, a topic at the intersection of computer science and statistics, makes it possible for computers to extract knowledge from data. This fusion of mathematical theory and algorithmic efficiency in computer science is necessitated by the demands of constructing statistical models from extensive datasets, often comprising billions or trillions of data points [2]. In the following sections, we provide a brief overview of the standard workflow for the development of ML models. Data preprocessing is an important step in the ML workflow and poses unique challenges in healthcare, owing to the need for clean and comprehensive datasets. The quality of data has a significant impact on the ability of a model to learn and generalize effectively. It is essential to gather representative data for training the model, adhering to the well-known principle of “garbage in, garbage out” [3]. Traditionally, many of these preprocessing tasks have been performed manually, but powerful data science applications have now emerged, accelerating these processes through automatization [4]. The development and validation of ML models involve a systematic approach using three distinct datasets. Initially, a training dataset is used, where the model learns by adjusting the weights to minimize the difference between the predicted and actual outcomes. Subsequently, a validation dataset aids in the model optimization and detection of overfitting. Finally, a test dataset is used to evaluate the generalization capabilities of the model and performance on new, unseen data. Cross-validation, a refinement of this approach, involves repeatedly dividing the data into different training and testing sets (or “folds”) to provide an average estimate of the model performance and reduce the risk of overfitting. This structured approach ensures that ML models are not only tailored to current data but are also robust and effective in predicting future outcomes [5]. In the ML field, methodologies are predominantly classified into supervised and unsupervised learning algorithms. Supervised ML is used for forecasting predefined outcomes and centralizing its approach to classification and prediction. These models operate by learning from labeled data, aiming to establish a robust relationship between input variables and a target outcome, such as classifying diagnoses [1,2,6]. In medical modeling, the generation of novel insightful features is more crucial than relying solely on existing predictors or advanced algorithms, as this is unlikely to lead to significant discoveries [2,4]. Unsupervised learning focuses on extracting structures and patterns from unlabeled datasets. This methodology is useful in identifying latent relationships and natural groupings within data. This approach is valuable in the context of precision medicine, aiming to re-conceptualize diseases based on underlying pathophysiological processes and facilitate the development of targeted therapeutic strategies. The effectiveness of these models is evaluated to assess their capacity to uncover biologically relevant patterns that inform diagnosis, treatment, and prognosis in kidney diseases [2]. Achieving high-performance unsupervised models requires not only the right features and model complexity but also a substantial volume of training data to avoid overfitting and ensure generalization to new, unseen cases. In clinical settings, this includes collecting extensive, unbiased data across multiple independent cohorts to validate the predictive power of the model [2,7].

**Figure 1:**
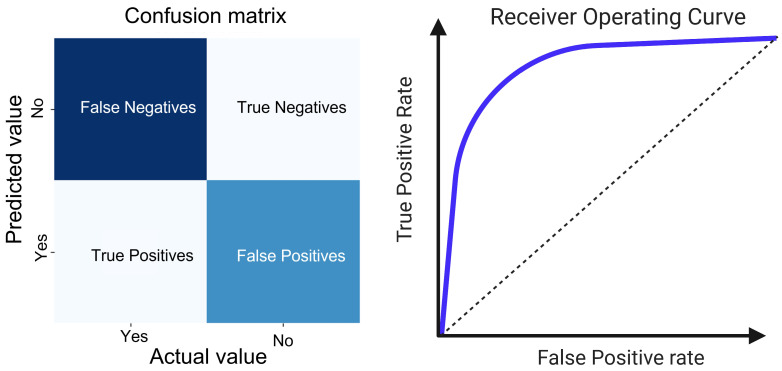
Classification metrics. The left section of the figure displays a confusion matrix, which is a tabular visualization of the predictive accuracy of the model. It categorizes the predictions into four distinct outcomes: true positives and true negatives, which represent the correctly identified instances of the two classes, and false positives and false negatives, which are erroneous predictions where the model incorrectly classifies the instances. The arrangement of these outcomes provides an immediate visual assessment of the classification process of the model, particularly in terms of its precision and recall capabilities. The ROC curve on the right serves as a graphical representation of the diagnostic ability of the model. It contrasts the true positive rate (sensitivity) with the false positive rate (1-specificity) across different thresholds. The diagonal dotted line represents the performance of a random classifier with an AUC of 0.5. The substantial elevation of the ROC curves of the model above this line of no-discrimination confirms the effective discriminative power between the two outcome classes.

In clinical practice, most clinicians encounter ML primarily through application of the latest research and guidelines. Thus, understanding commonly used performance metrics for evaluating ML algorithms is more essential than a deep knowledge of the inner workings of the algorithm. Although ML algorithms offer significant benefits, they are not without limitations. A particularly crucial challenge is balancing overfitting and underfitting within the datasets. An ideally fitted model demonstrates strong performance on the training, validation, and test datasets. It is important to note that the quality of any predictive model is fundamentally limited by inherent signals in the input datasets. This constraint can be mitigated through orthogonal validation using independent datasets or different data modalities, which can further corroborate ML-driven feature selection and predictive analysis [8]. Model evaluation can vary but often focuses on prediction accuracy, F1-score, sensitivity, specificity, and area under the ROC curve (AUROC) (Figure 1) [9]. We now describe the selection of ML models commonly employed in the field of clinical medicine.

### 1.1. Linear Regression

Linear regression is employed to establish a connection between the numeric features and a single numeric target. This technique employs a linear model, typically represented as y = ax + b, in univariate scenarios, where ‘a’ signifies the slope dictating the change in the *y*-axis per unit increase in x, and ‘b’ denotes the *y*-axis intercept. Multivariate linear regression extends this concept to include multiple features and their respective weights. In practice, perfect fitting is rare, and the error in the fit is quantified using residuals, which are deviations between the predicted and actual values [9,10,11]. Linear regression models could enhance the prediction of disease progression and estimate the glomerular filtration rate (GFR) in nephrology. They may enhance personalized care and preventive strategies for patients progressing to chronic kidney disease.

### 1.2. Logistic Regression

Logistic regression is a classification algorithm that aims to establish a connection between the features and the probability of a specific outcome. Unlike linear regression, which uses a straight line, logistic regression employs a sigmoidal curve characterized by a sigmoid function to estimate class probabilities. This curve transforms discrete or continuous numeric features into numerical values within a range of 0 to 1. The key advantage of this approach is that it ensures that probabilities remain suitable for binomial (two possible outcomes) scenarios and is extensible to multinomial (three or more possible outcomes) scenarios [9,10,11]. This statistical method may be beneficial in developing risk assessment models for kidney disease progression and in identifying patients at a high risk of developing complications.

### 1.3. Decision Trees

ML decision trees operate by creating a tree-like decision structure, where each node represents a feature or attribute, while the branches represent decision rules. Interpreting decision trees involves tracing the path from the root to a leaf node following the decision criteria at each node. This interpretability is one of the key strengths of decision trees, as it allows clinicians to understand the decision-making process, making them more transparent and explainable than many other ML models. However, a significant limitation is the tendency towards overfitting, particularly with complex or noisy datasets [9,10,11]. Decision trees in nephrology can classify patients into different risk categories based on their clinical and demographic characteristics, offering a visual and interpretable model for diagnosing and predicting kidney disease progression.

### 1.4. Random Forest

A random forest operates by creating multiple decision trees during the training phase and then generates outputs based on the mode (in classification) or mean prediction (in regression) of these trees. This method effectively amalgamates the outputs of individual trees to yield accurate and stable predictions. While each tree in the forest is straightforward, the collective model is often seen as a ‘black box’ because of the integration of the decisions of multiple trees. However, interpretation is aided by using feature importance scores, which shed light on the most influential variables in the predictions of the model. The random forest strikes a balance between accuracy and robustness in the analysis of clinical data [9,10,11]. It can enhance diagnostic and prognostic accuracy in kidney disease through its ability to handle complex interactions and nonlinear relationships among variables.

### 1.5. KNN

The k-nearest neighbor (k-NN) algorithm operates by measuring the similarity between data points and relies on the proximity of neighbors in a training dataset to make predictions for new data. The choice of “k”, the number of neighbors considered, is crucial. Smaller k values lead to flexible models, whereas larger values lead to smoother predictions. However, k-NN has several drawbacks. It can be mathematically intensive, especially with large datasets, and its performance is sensitive to the choice of k, which, if poorly selected, can lead to reduced accuracy. In addition, k-NN lacks memory, requiring an algorithm to reevaluate previously seen data [4]. It can aid in the diagnosis and prognosis of kidney disease by classifying patients based on the similarity of their clinical features with those of known cases.

### 1.6. SVM

Support vector machines (SVMs) represent a powerful class of supervised ML algorithms employed for tasks involving classification and regression. The primary objective of SVMs is to ascertain an optimal decision boundary, that is the hyperplane, which effectively segregates data points into distinct categories or predicts continuous values. The fundamental objective is to identify the hyperplane that optimally augments the margin. SVMs classify new unseen data points by determining their positioning with respect to the hyperplane [4]. The ability of a SVM to handle high-dimensional data makes it suitable for prognostic modeling, predicting disease progression, and treatment outcomes, thereby aiding the development of tailored therapeutic strategies.

### 1.7. ANN

An artificial neural network (ANN) is an ML algorithm inspired by biological neural networks. In ANNs, nodes (like the cell bodies in biological neurons) communicate through connections (akin to axons and dendrites). These transformations ultimately lead to the representation of inputs predictive of the desired outcome. The choice between traditional ML techniques (e.g., Random Forest) and ANNs depends on the nature of the task and data, with traditional ML techniques offering transparency and simplicity for structured data, whereas ANNs excel in capturing intricate patterns within unstructured data but require greater resources. For image recognition, traditional feedforward ANNs process each pixel individually, thereby losing the spatial context. Convolutional neural networks (CNNs) overcome this problem by feeding image patches to specific nodes in the next layer, thereby preserving the spatial relationships. When a neural network consists of two or more hidden layers, it is called Deep Learning (DL). Beyond image classification, DL has shown potential for image-segmentation tasks. Rather than classifying entire images, this approach aims to identify objects within an image by classifying individual pixels based on information from surrounding pixels. For instance, in diabetic retinopathy, a segmentation algorithm can outline the retinal vasculature by assigning probabilities to each pixel, indicating whether it belongs to a retinal blood vessel. A similar approach can be used for breast cancer detection by marking pixels as part of a mass, allowing radiologists to further examine the output image [9,10,11]. Their ability to learn from vast datasets enables the identification of subtle patterns associated with disease progression and patient responses to treatment, thereby facilitating personalized and predictive nephrology care.

## 2. Current Status of Machine Learning in CKD

Recent advancements in ML have opened up new horizons for precision medicine in the field of nephrology. The Nephrotic Syndrome Study Network (NEPTUNE) is at the forefront of applying precision medicine by formulating novel disease definitions via detailed, multi-layered analysis within observational cohort studies. Despite being at an early stage, this approach holds promise for the future, particularly in renal pathology research and forecasting the risk of kidney diseases or renal function deterioration [12,13]. The deployment of ML in biological image analysis has been robust and swift and it has established itself as a dependable technique for identifying malignancies [14,15,16,17,18,19]. In the field of nephrology, ML-based biological image analysis has shown promise in diagnosing renal pathology and is considered the definitive standard for identifying kidney diseases. This diagnostic approach directly influences the spectrum of treatment choices and patient outcomes. Traditional glomerular evaluation methods (light microscopy, morphometric analysis, and electron microscopy) are still manual, time-consuming, and lack standardization. Consequently, recent initiatives have been aimed at automating glomerular injury quantification [20]. Initially, Marée et al. [21] demonstrated a supervised learning approach for detecting glomeruli, achieving a model with 95% precision rate and 81% recall. This segmentation serves as the foundational step in automated renal pathological diagnosis, enabling the precise recognition of glomerular and tubular structures within renal pathological imagery. Preliminary investigations revealed that utilizing AI greatly improved the accuracy of the estimated glomerular filtration rate (eGFR) [22]. Several studies collectively highlight the successful application of AI and ML in improving the prediction and management of CKD [23,24,25]. Tangri et al. [23] demonstrated a highly accurate Cox proportional hazards model for predicting kidney failure by utilizing standard laboratory data from electronic health records. Norouzi et al. [24] advanced this approach by using a neuro-fuzzy system and a decade of clinical data to accurately predict kidney failure timelines in patients with CKD. Perott et al. utilized an unsupervised learning method to effectively predict the transition from CKD stages III to IV. These studies collectively suggest that AI and ML, through various approaches, can significantly enhance the accuracy of CKD prognosis and management, marking a promising direction for future research and applications in nephrology.

### 2.1. Diabetic Kidney Disease

Diabetes mellitus (DM) is the primary cause of kidney failure in the Western hemisphere [26,27,28]. The initial clinical sign of diabetic kidney disease (DKD) is typically the emergence of microalbuminuria, defined as an excretion of ≥30 mg/day or 20 µg/min. However, kidney biopsy studies indicate that microalbuminuria is not exclusively indicative of type 2 DKD, as only 20–40% of patients with this condition progress to apparent kidney disease without targeted treatment. Conversely, approximately 20% of individuals with type 2 DM maintain normal urinary albumin levels while advancing to CKD stage 3, characterized by a GFR below 60 mL/min/1.73 m^2^. This underscores the critical need for the development of new non-invasive biomarkers that can more accurately detect the early stages of DKD and forecast the progression to renal impairment or kidney failure [26].

First, in individuals with DM and kidney disorders, it is crucial to accurately distinguish between DKD and non-diabetic kidney disease (NDKD) to direct treatment strategies and enhance patient outcomes. The meta-analysis conducted by Liang et al. [29] highlighted that the lack of diabetic retinopathy, a brief duration of diabetes, along with lower levels of HbA1c and systolic blood pressure, serve as relatively effective indicators for differentiating NDKD from DKD [29]. Although numerous markers have been identified in the literature as significant for differentiating DKD from NDKD, establishing a method to accurately, safely, and scientifically diagnose DKD remains a challenge. Kidney biopsy is the most definitive approach for diagnosing DKD. However, not all patients are suitable candidates for this procedure for various reasons, including anticoagulation therapy, previous unilateral nephrectomy, or reluctance to undergo a biopsy. Utilizing logistic regression analysis based on five key differential indicators (duration of DM, systolic blood pressure, HbA1c levels, presence of hematuria, and diabetic retinopathy), Zhou et al. [30] tried to create a differential diagnostic model (Table 1). Their AUC of 0.968 nearly reached the ideal score of 1.0, indicating an exceptional predictive capability. Subsequent testing on a validation cohort maintained impressive results, with a sensitivity of 80% and specificity of 100%, underscoring the promising predictive potential. Using electronic health records, deep feature learning, and supervised classification techniques, numerous diabetes studies have been able to forecast diabetes occurrence and identify contributing risk factors [31]. Given the significant influence of diabetic complications on quality of life and mortality rates, a multitude of researchers are dedicated to exploring the use of ML to enhance the diagnosis and management of these diabetic complications [20]. In a pioneering study by Cho et al. [32], medical data from 4321 diabetic patients over a 10-year period were used to create a support vector machine classification-based visualization system. This model could predict the onset of DKD with high accuracy (AUC of 0.969), approximately 2 to 3 months before clinical diagnosis. Although not entirely accurate, it marked the first use of data mining and advanced ML algorithms for predicting DKD, offering a novel approach to early-stage treatment strategies. Several ML algorithms using data from 10,251 patients from the ACCORD trial were generated to model the risk of DKD. The study highlighted that the random forest and logistic regression models outperformed others in prediction accuracy, with sensitivity and accuracy exceeding 0.72 and 0.73, respectively, for random forest, and 0.76 and 0.8, for logistic regression. A decrease in eGFR was identified as a significant indicator of DKD progression. Moreover, these ML models identified additional indicators such as creatinine phosphokinase, fasting plasma glucose, and potassium. It also recognized shifts in fasting plasma glucose and changes in GFR after the first year as early and subsequent biomarkers of DKD, respectively [33]. However, these models did not undergo external validation. Further advancing the field, a logistic regression-based Roche/IBM algorithm and real-world data to predict CKD onset in patients with DM achieved an AUC of 0.794. The performance of this model was better than that of large randomized controlled trials. Data from 522,416 patients were used for model building and cross-validation, with additional external validation from the data of 82,912 patients from the Indiana Network for Patient Care database [34]. Adding a genetic dimension, ML models alongside phenotypic features significantly improved the prediction accuracy to 0.9 [35]. Age, age at diagnosis, and lipid levels have emerged as key clinical indicators, whereas genetic polymorphisms associated with inflammation and lipid metabolism are among the genetic predictors. Validating these genetic markers in individuals without clinical signs of kidney disease could pave the way for identifying high-risk individuals. This would facilitate regular monitoring and personalized management strategies, particularly focusing on controlling inflammation and dyslipidemia, as proactive measures to prevent DKD.

### 2.2. IgA Nephropathy

IgA nephropathy (IgAN), also known as Berger’s disease, is the most common form of primary glomerulonephritis worldwide. Its epidemiology varies geographically, with a higher prevalence in Asia and Southern Europe. It typically presents in the second and third decades of life and more often affects males [36]. Clinically, IgAN is characterized by the presence of IgA-dominant immune deposits in the glomerular mesangium, leading to variable clinical presentations [37,38]. The hallmark symptom is hematuria, which may be episodic and often coincides with upper respiratory tract infections. Proteinuria is also common, ranging from mild to nephrotic. Hypertension and edema may occur, particularly in advanced cases [37]. The diagnosis is primarily based on kidney biopsy, which reveals mesangial proliferation and IgA deposition. Supportive diagnostic markers include elevated serum IgA levels and presence of IgA-containing immune complexes. Kidney function tests and imaging are used to assess the extent of renal impairment [39]. Geddes et al. [40] demonstrated that an artificial neural network, when trained with standard clinical data collected at diagnosis, had the potential to predict kidney function decline over a 7-year period in patients with IgAN more precisely than seasoned nephrologists. The application of ML algorithms to predict kidney failure progression in IgAN patients has evolved significantly over the years [40,41,42,43]. In an early attempt in 1998, an artificial neural network was employed to create a predictive model for kidney failure progression in patients with IgAN, achieving a sensitivity of 86.4% and specificity of 87.5%. Despite outperforming experienced nephrologists in predicting kidney function decline, the limitation of this model was its small sample size of 54 patients and lack of validation [40].

Although as many as 40% of IgAN patients may develop kidney failure, determining which patients will progress remains a challenge. A convenient and efficient approach that can accurately predict long-term kidney prognosis would be highly beneficial for nephrologists, enhancing their ability to make informed clinical decisions [37]. The absolute renal risk (ARR) model is a well-known prognostic tool for predicting kidney failure or death in IgAN patients. It utilizes factors, such as hypertension, proteinuria ≥1 g/24 h, and severe histopathological renal lesions [44]. However, it does not consider the initial eGFR or serum creatinine, which is known to be a significant indicator of renal outcomes in IgAN. An artificial neural network model has shown enhanced predictive accuracy for IgAN compared to the artificial neural network model. This artificial neural network model was converted into an online clinical decision-support system, designed for quantitative risk assessment of kidney failure and its timing in IgAN patients, factoring in six predictors, including initial serum creatinine. Notably, eGFR was not incorporated into the model. While the artificial neural network model was trained on diverse population cohorts, the representation of the Asian population was smaller. This discrepancy suggests that the universal applicability of the clinical decision support system, particularly in Asian patients, may require additional validation and consideration [41]. Advancements in data handling and ML have led to the development of robust artificial neural network models. This model was integrated into an online clinical decision support system for patients with IgAN to provide quantitative risk assessments and time predictions for kidney failure. The model, which relied on three variables (urine protein excretion, global sclerosis, and tubular atrophy/interstitial fibrosis) yielded a C statistic of 0.84 (with a 95% confidence interval of 0.80–0.88). The risk stratification ability of the statistical shape modeling was further validated through Kaplan–Meier analysis, which clearly identified significant risk differences among the patients [45]. Other ML approaches have been explored, showing that the random forest model was superior in predicting kidney failure progression, with a sensitivity of 80.6% and a specificity of 95.3% [37,39]. Liu et al. [39] further refined the random forest model to achieve an impressive AUC of 97.3% by including the C3 staining results, demonstrating the utility of the random forest algorithm in staging and managing various progressive diseases. However, the focus of current research is predominantly on predicting kidney failure in patients with existing IgAN, and there is a gap in modeling risk prediction of developing IgAN, which may be an avenue for future research.

### 2.3. Hemodialysis

ML is being increasingly utilized across a wide spectrum of activities in dialysis treatment, ranging from prescription and monitoring to managing complications and predicting patient mortality, and holds significant potential in the domain of pediatric dialysis [46,47,48]. Notable insights into the expansive use and promising future of AI in dialysis were shared at a pioneering scientific conference at the Hospital Universitari de Bellvitge, where discussions revolved around deployment, challenges, and prospective advancements of AI in the dialysis sector [49]. Regarding prescription management, the challenge of defining and measuring dialysis adequacy is also being addressed by AI [46]. Techniques such as direct dialysate quantification (DDQ) for assessing urea nitrogen (UN) removal provide accurate real-time data on urea removal. Urea kinetic modeling is a method used in nephrology to assess and individualize dialysis treatment in patients undergoing hemodialysis or peritoneal dialysis. In this way, it offers the additional benefit of predicting the necessary dialysis dose for achieving the desired treatment goals, making it a valuable tool for personalized patient care. Advances in AI, particularly neural networks, have enhanced the prediction accuracy of solute concentration fluctuations and UN removal rates during and post hemodialysis sessions [50,51]. The predictive neural network model developed by Akl et al. [46] demonstrated no substantial discrepancy from the actual time intervals needed to achieve the same solute removal index under identical patient conditions (such as blood urea nitrogen concentration and patient weight). The only exception was in predicting symptomatic rapid intradialytic during the initial 30 min interval of dialysis, where a notable difference was observed (*p* = 0.001). This deviation arises because following the first 30 min of dialysis there is a consistent time delay before the equilibrated urea concentration (i.e., the average concentration across the body) aligns with the blood concentration. Nonetheless, larger and more comprehensive datasets are necessary for model optimization.

Anemia is a prevalent complication in dialyzed patients, presenting a multifaceted challenge for nephrologists [52]. Numerous strategies have been implemented aimed at achieving accurate hemoglobin predictions [47,49,52,53,54,55,56,57]. Although erythropoiesis-stimulating agents (ESAs) have been effective in treatment, the variability in patient response necessitates a personalized approach to dosing. To assist nephrologists in determining the dosages of ESAs and iron, an AI-based tool was created. The anemia control model is composed of two parts: (1) an artificial neural network model that forecasts future hemoglobin levels based on current clinical data from the patient and (2) an algorithm that proposes the most suitable ESA and iron dosages to meet the hemoglobin target. It is crucial to note that the anemia control model offers treatment suggestions and requires validation. It has shown efficacy in not only managing but also improving treatment outcomes [52,54,58]. The implementation of the anemia control model in clinical settings has resulted in an increase in the percentage of patients achieving their target hemoglobin levels (from 70.6% to 83.2%), a decrease in hemoglobin fluctuation (from 0.95 to 0.83 g/dL), a marked decrease in instances of hemoglobin levels exceeding 12 g/dL (from 18.1% to 7.5%), and a reduction in the usage of ESA and iron. However, challenges persist in precise dose determination, underscoring the complexity of treatment personalization.

**Table 1:** Overview of the studies investigating several machine learning methods in diabetic kidney disease, IgA nephropathy, and hemodialysis patients. Abbreviations: DN: Diabetic Nephropathy; NDRD: Non-Diabetic Renal Disease; AUC: Area Under the Curve; DM: Diabetes Mellitus; CKD: Chronic Kidney Disease; IgAN: IgA Nephropathy; ESKD: End-Stage Kidney Disease; eGFR: estimated Glomerular Filtration Rate; SRI: Solute Removal Index; DDQ: Direct dialysate quantification; Hb: Hemoglobin; MAE: Mean Absolute Error.

Disease	ML Model	Dataset	Outcome	Metrics	Bottlenecks	Data Availability	Reference
DKD	Logistic regression	110 patients: 60 DN patients and 50 NDRD patients	DN vs. NDRD	Sensitivity of 90% Specificity of 92% AUC of 0.968	Monocentric study. Less effective for non-linear problems. Requires careful feature selection.	No data availability	[30]
	Support vector machines	4321 diabetic patients	Predict DN	AUC of 0.969	Difficult to tune hyperparameters. Not ideal for very large datasets.	No data availability	[32]
	Random forest	10,251 patients	Risk of DN	Sensitivity 72% Accuracy of 73%	No external validation. Can lead to overfitting in complex models. Interpretation can be challenging.	Data available	[33]
	Logistic regression	10,251 patients	Risk of DN	Sensitivity of 76% Accuracy of 80%	No external validation. Less effective for non-linear problems. Requires careful feature selection.	Data available	[33]
	Logistic regression	522,416 DM patients in the training set and 82,912 DM patients in the validation set	Predict CKD	AUC of 0.794	Less effective for non-linear problems. Requires careful feature selection.	No data availability	[34]
	Support vector machines	119 subjects with DN and 554 without DN at enrolment	Predict DN	Accuracy of 95.0%	Difficult to tune hyperparameters. Not ideal for very large datasets.	Limited data availability	[35]
	Random forest	119 subjects with DN and 554 without DN at enrolment	Predict DN	Accuracy of 90.0%	Can lead to overfitting in complex models. Interpretation can be challenging.	Limited data availability	[35]
IgA nephropathy	Random forest	262 Asian IgAN patients	Predict ESKD	AUC of 97.29% F-score of 83.0%	Can lead to overfitting in complex models. Interpretation can be challenging.	Limited data availability	[39]
	Artificial neural network	54 patients with IgAN	Predict outcome at 7 years	Accuracy of 87.0% patients Sensitivity of 86.4% Specificity of 87.5%	Lack of validation. Small sample size. Requires substantial computational resource. Model complexity can obscure interpretability.	No data availability	[40]
	Artificial neural network	1040 biopsy-proven IgAN patients (from Italy, Norway, and Japan)	Risk and timing of ESKD	AUC of 0.899–1.000 in different geographic cohorts F-score of 70.8–90.7% in different geographic cohorts	Requires substantial computational resources. Model complexity can obscure interpretability.	Data available	[41]
	Decision tree	790 Japanese IgAN patients	Risk stratification for renal deterioration	AUC of 0.830 (95% confidence interval, 0.777–0.883)	Prone to overfitting. Simplicity can lead to less nuanced decisions.	Limited data availability	[43]
	Logistic regression	790 Japanese IgAN patients	Risk stratification for renal deterioration	AUC of 0.808 (95% confidence interval, 0.754–0.861)	Less effective for non-linear problems. Requires careful feature selection.	Limited data availability	[43]
	Gradient boosting	Validation cohort of 1025 patients with IgAN from 18 renal center	Combined event of ESKD or 50% reduction in eGFR	C-statistic of 0.840 (95% confidence interval, 0.80–0.88)	Sensitive to overfitting with small datasets. Long training times.	Limited data availability	[45]
Hemodialysis	Artificial neural network	15 chronic hemodialysis patients	Predict hemodialysis session time needed to reach a target SRI in patients	Prediction error of 10.9% (compared to DDQ model)	Requires substantial computational resource. Model complexity can obscure interpretability.	Limited data availability	[46]
	Random forest	27,615 US veterans with incident ESKD	Risk of death	C-statistic of 0.7185 (95% confidence interval: 0.6994–0.7377) C-statistic of 0.7446 (95% confidence interval: 0.7346–0.7546) C-statistic of 0.7504 (95% confidence interval: 0.7425–0.7583) C-statistic of 0.7488 (95% confidence interval: 0.7421–0.7554) for predicting risk of death 30-, 90-, 180-, and 365-day all-cause mortality after dialysis initiation, respectively	Can lead to overfitting in complex models. Interpretation can be challenging.	Limited data availability	[48]
	Artificial neural network	52 patients undergoing hemodialysis therapy	Hb fluctuation	The percentage of Hb values within target range increased from 70.6% to 76.6%	Requires substantial computational resources. Model complexity can obscure interpretability.	No data availability.	[52]
	Artificial neural network	688 patients (21,866 records) from Italian clinics, 1397 patients (24,565 records) from Spanish clinics, and 2050 patients (55,487 records) from Portuguese clinics formed part of this study	Hb prediction	MAE ranging from 0.548 to 0.613 across different countries	Requires substantial computational resources. Model complexity can obscure interpretability.	No data availability.	[54]
	Random forest	826 hemodialysis patients of 66 years or older	Sudden cardiac death the day of or day after a dialysis session	C-statistic of 0.799 including both predialysis and postdialysis information	Can lead to overfitting in complex models. Interpretation can be challenging.	No data availability.	[59]

Models developed for short-term mortality prediction post-dialysis show good predictive reliability, although the need for broader external validation is recognized [48,59,60]. In a large study involving 27,615 U.S. veterans newly diagnosed with kidney failure, a random forest model yielded C-statistics with 95% confidence intervals of 0.7185 (0.6994–0.7377), 0.7446 (0.7346–0.7546), 0.7504 (0.7425–0.7583), and 0.7488 (0.7421–0.7554), corresponding to the ability to predict mortality risk within four distinct time frames (30, 90, 180, and 365 days after beginning dialysis). These models demonstrated solid internal consistency and were effectively replicated across patients with various demographic and clinical backgrounds. They offer comparable or superior predictive performance relative to other ML algorithms. However, the applicability of these results beyond the veteran population may be limited. Moreover, reliance on electronic medical records for predictor variables constrains the breadth of the predictor assessment. These models utilize the characteristics of patients before dialysis, potentially assisting in the decision-making process before starting dialytic treatments. They provide patients and health care professionals with an understanding of the expected short-term mortality risk associated with choosing this treatment option. Having a quantifiable risk assessment could simplify the decision-making process for patients when comparing dialysis with other treatment options such as medication-only approaches or palliative care. This could lead to improved outcomes centered on the quality of life of the patients, particularly for those with a high risk of short-term mortality, and might also contribute to healthcare cost savings [48].

Moreover, the utility of ML in predicting sudden cardiac death, a critical risk for dialysis patients, further exemplifies its potential. A robust random forest model was established using the data available before the start of the dialysis session (C-statistic 0.782). The accuracy of this model was modestly enhanced when it incorporated data gathered during and after the dialysis session, reaching a C-statistic of 0.80 in the validation dataset (*p* < 0.001). Intriguingly, the key indicators for short-term predictions are metrics that vary considerably between dialysis sessions, such as blood pressure and changes in weight during dialysis. In contrast, serum albumin stands out as an exception, given its relative stability over time. However, the accuracy of the model decreased over longer time ranges [59]. The predictive capacity of ML models offers valuable insights for early clinical decision making, enhancing patient care and treatment strategies.

## 3. Future Prospects of Machine Learning in CKD

While most nephrologists currently lack familiarity with the fundamental principles of medical AI, there is a promising future potential for collaborative efforts between nephrologists and AI researchers. This collaboration could lead to the creation of an extensive database for CKD research and development of a highly efficient model for CKD diagnosis and treatment. One notable initiative involves Google DeepMind and the U.S. Department of Veterans Affairs, culminated in the creation of an AI system capable of predicting AKI up to 48 h before its clinical manifestation. By leveraging electronic health record (EHR) data, this system exemplifies how integrating data science into nephrology can yield practical tools for the early detection and prevention of kidney damage, potentially saving lives and optimizing resource allocation in healthcare [61].

The intricacy of pathological presentations of different kidney diseases and their tight interaction with clinical indicators have led to the rapid investigation of AI. Nonetheless, the automated pathological diagnosis of kidney diseases based on photographs has not yet been documented. With thorough patient information, pathologists cannot fully use it as a substitute for the renal pathological diagnosis of all types of kidney disorders. This needs to be supported by a large amount of data and verified by future research. To confirm this technique in the distribution and spectrum of all lesions found in typical renal pathology services, additional validation of the DKD framework and image processing across various clinical practices and image datasets is required [20]. Through advancements in clinical data storage and processing technology, and the establishment of a resource-sharing platform, kidney disease risk models based on extensive multicenter data may become more dependable. When these models achieve a sufficiently high level of performance, they may eventually replace the need for kidney biopsy, enabling noninvasive diagnoses. While the future remains uncertain, it appears that these developments are increasingly likely [20].

**Figure 2:**
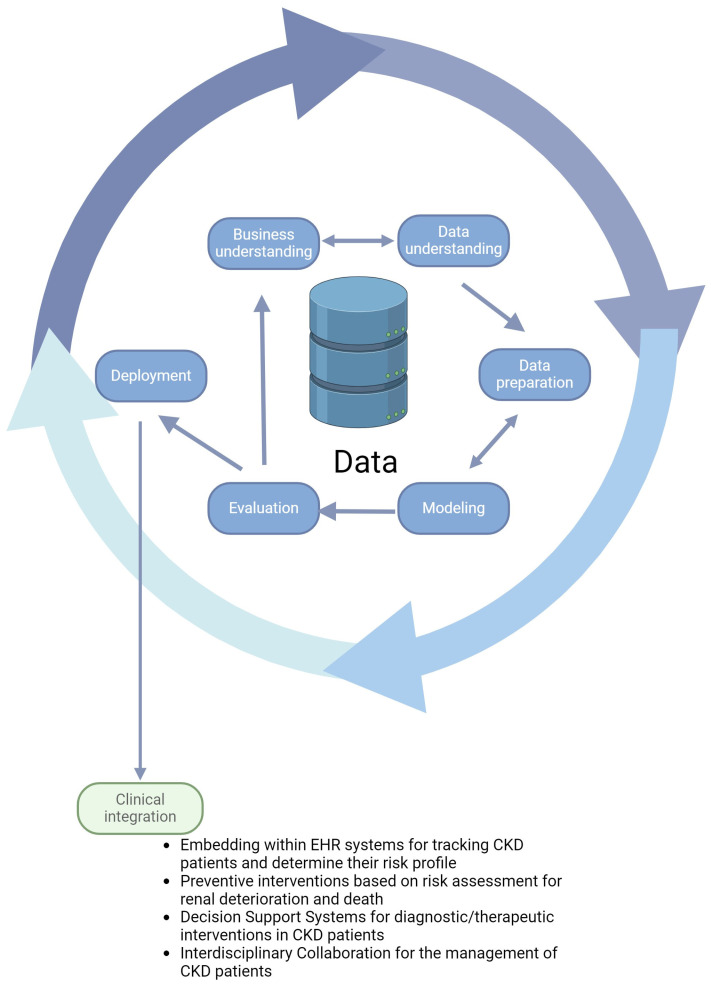
Overview of the standard machine-learning workflow with its integration in clinical practice. The Cross-Industry Standard Process for Data Mining (CRISP-DM) is a robust cyclic methodology that guides the data-mining process. The process initiates with the business understanding phase, which is dedicated to grasping the business aspects of the project and translating them into a data analytical framework. Progressing to data understanding, this stage involves an initial acquisition of data followed by an exploration to familiarize itself with the data characteristics, quality, and potential of the datasets. During this phase, preliminary insights are gleaned and the groundwork for hypothesis formation is laid. Subsequently, data preparation constitutes a critical phase in which raw data undergo transformation and cleansing, ensuring that they are primed for analysis. The modeling phase sees the application of various algorithmic techniques, fine-tuned to model the data effectively on the training and validation set. In the evaluation phase, the models and the entire process undergo scrutiny to ascertain their efficacy in meeting the defined business objectives on the test set. Finally, the deployment phase represents the culmination of the process, where the insights and findings are operationalized [64]. Next, the ML model can be integrated into clinical workflows. These models must be seamlessly incorporated into existing clinical workflows to augment clinical decision making and effectively enhance CKD patient management. This involves embedding the ML model interface within electronic health record (EHR) systems, allowing clinicians to access predictive insights alongside comprehensive patient data without disrupting routine practices. As part of a clinical decision support system (CDSS), the ML model provides clinicians with predictions of CKD progression, identifying patients at high risk of advancing to kidney failure and suggesting potential interventions. These can range from medication adjustments and dietary modifications to discussions about initiating dialysis, enabling earlier and more targeted interventions with the potential to slow disease progression, improve patient outcomes, and reduce healthcare costs. This iterative process is supported by interdisciplinary collaboration that brings together nephrologists, data scientists, IT specialists, and patients to align the model with clinical needs and patient care goals.

However, integrating AI into healthcare, particularly nephrology, presents unique challenges. Data drift, where the input data evolve over time and diverge significantly from the initial training data, poses a significant challenge. Active or continuous learning methods can enable AI models to adapt and improve. The adoption of Machine Learning Operations (MLOps) is critical for automating deployment processes and ensuring the ongoing maintenance of ML models, thereby supporting a data-driven approach to healthcare [4]. Safeguarding patient confidentiality necessitates adherence to established ethical frameworks and regulations such as the General Data Protection Regulation (GDPR) in Europe and the Health Insurance Portability and Accountability Act (HIPAA) in the United States. These frameworks mandate strict data anonymization processes, patient consent for data usage, and the establishment of data sharing agreements that ensure confidentiality and security. Ethically, it is crucial to balance the potential benefits of ML in nephrology with respect for patient autonomy and privacy and the minimization of potential harm. The Belmont Report’s principles of respect for persons, beneficence, and justice provide a foundational ethical guideline for conducting research that involves patient data, emphasizing the need for transparency, patient consent, and equitable access to the benefits of research advancements. Additionally, the probabilistic nature of ML necessitates setting predefined thresholds of acceptable errors before engaging in ML projects. The complexity of some ML algorithms makes it challenging for physicians to interpret results, highlighting the importance of Explainable AI (XAI). XAI provides transparency in AI decision-making processes, fostering trust and ensuring compliance with regulations such as GDPR. XAI aids in identifying and correcting biases and errors in AI systems, ensuring ethical, fair, and safe operations. It enhances human–AI collaboration by making the actions of AI systems understandable and predictable for their human users. Clinicians traditionally exercise caution in uncertain diagnostic scenarios, a practice that is not mirrored by machines. This discrepancy underscores the importance of clinicians being well versed in AI technologies, recognizing their limitations, and maintaining patient safety. Examples such as the limitations of pulse oximetry devices in detecting carbon monoxide poisoning illustrate the potential pitfalls of overrelying on automated systems without understanding their limitations [1,62]. Furthermore, mitigating biases in datasets and ensuring the generalization of ML models is important. Firstly, the collection of data from a broad spectrum of demographics, healthcare settings, and geographic locations is imperative. Secondly, the identification and quantification of biases within datasets through statistical techniques and data visualization are essential. The application of fairness metrics, including equality of opportunity, predictive parity, and demographic parity, serves as a third step. These metrics facilitate the evaluation of the performance of ML models across various groups, guiding the adjustment of models to enhance fairness. Fourthly, external validation of ML models on datasets from diverse populations and settings is crucial for assessing generalizability. This process helps identify and rectify biases that were not evident during the initial training phase. By implementing these steps, researchers and practitioners in nephrology can advance the development of ML models that are not only scientifically robust but also ethically responsible and inclusive, thereby maximizing the potential benefits of ML in improving healthcare outcomes across diverse global populations. To enhance the development of guidelines and policy implementation in public health and medicine through the synthesis of evidence from multiple studies and meta-analyses, there is a need for a new approach to disseminating and reporting ML models in medicine. This approach should include the provision of all model parameters, the use of standard ontologies to describe variables, and the disclosure of data transformations and sampling methods. Collaboration between clinicians and data scientists is crucial for defining data-sharing and usage policies, aiming to improve the generalizability and credibility of ML algorithms in nephrology [63]. Figure 2 shows an example of how ML models are designed and how they can be valuable in CKD progression.

## Data Availability

Not applicable.
